# The Influence of Deep Space and the Stars on Emotions

**DOI:** 10.1002/ijop.70146

**Published:** 2025-12-11

**Authors:** Jason P. Martens, Mia Prokopetz, Kit Tomlinson

**Affiliations:** ^1^ Department of Psychology Capilano University North Vancouver Canada

**Keywords:** awe, James Webb telescope, restoration, stars, urban

## Abstract

Detailed photographs of deep space from the James Webb telescope are public, yet little is known about how such imagery might affect people. Using both face‐to‐face and online study designs, compared to exposure to photographs of urban environments, exposure to photographs of deep space and stars increased experiences of awe overall and also its 6 subfactors, and in particular vastness (e.g., “I felt in the presence of greatness”) and accommodation (“I found it was hard to comprehend the experience in full”). Effects were generally larger for photographs of deep space than those of the stars. Mixed results were found on positive affect in general, with it sometimes increasing after exposure to deep space and the stars. No effects emerged on negative affect. Deep space and stars also led to higher ratings of pleasantness of the images, perceived restoration, and willingness to hang such photos in their room compared to urban photographs. Moderators were also assessed (i.e., feeling connected to the night sky and fear of the dark). Overall, results suggest that photographs of deep space from the James Webb telescope have similar, though not identical, effects as photographs of stars, both of which are generally more positive than urban photographs.

## 1 Introduction

Growing research suggests that exposure to natural environments can have positive effects on emotions (e.g., McMahan and Estes 2015). Whether natural environments are green spaces (e.g., wilderness; Hartig et al. 1991), blue spaces (e.g., beaches; White et al. 2010), or even “dark spaces” (e.g., the night sky; Smalley and White 2023), such environments generally seem to leave people emotionally better off than when exposed to human‐made, urban, grey spaces (e.g., cities). However, dark spaces have received relatively little empirical work, yet stars and the night sky have prominently influenced cultures from around the world (Penprase 2017), and new technologies have exposed people to photographs of deep space environments in a way that has not been previously possible, such as the James Webb telescope that has provided awe‐inspiring photographs (Dimitrova 2024). How do such photographs affect people?

Broadly, astronauts report feeling intense emotional experiences that include awe and transcendence when looking back at Earth from space (Yaden et al. 2016), and qualitative reports of undergraduates in a space simulator are mostly similar to those of astronauts (Gallagher et al. 2014). Similarly, when members of an astronomy group stargaze, they tend to feel more positive emotions like awe and wonder, and also instances of transcendence (e.g., feeling small in the universe, losing sense of time; Bell et al. 2014). Tourists who observe the night sky (i.e., astrotourists) also tend to experience awe, which is related to being more satisfied with their visit and intention to return (Rodrigues et al. 2022). Participants who observed a simulated view of Earth from space reported more awe using a single‐item measure compared to baseline, but no effect of space was found on positive or negative affect (Chirico et al. 2018). Although awe is typically experienced positively and is associated with increased well‐being (Rudd et al. 2012), when participants are shown a short video of the Earth and stars, both positive and negative experiences of awe are elicited (Gordon et al. 2017).

Conversely, night skies are not always associated with awe. When features are systematically varied in an urban and natural setting using computer‐generated images, nighttime scenes where stars and the moon are present (in addition to the surrounding environment, such as city landscape and lake) produced some of the lowest scores on self‐reported awe using a single‐item measure (Smalley and White 2023). Taken together, research generally suggests that dark spaces are related to feelings of awe, but not always.

There are gaps in our understanding of dark spaces. Studies often use a self‐selected sample using qualitative data. There are limits in the studies using an experimental design, such as using simulated images rather than genuine ones. Studies often rely on single‐item measures of awe that do not capture awe's complexity, and there are potential moderators that have not been assessed.

We aimed to address these gaps in the literature by recruiting a diverse sample of participants (rather than self‐selecting) and employing a within‐subjects experimental design of actual (not simulated) photographs. We used a previously validated multi‐faceted measure of awe that contains 6 subscales (Yaden et al. 2019), which more fully captures awe experiences than single‐item measures. We also assessed two potential moderators. The first was how connected people feel to the night sky using the recently developed night sky connection index (NSCI; Barnes and Passmore 2024), as such a connection might lead to more positive experiences when observing dark spaces. Limited research exists on the NSCI given its recent development, but among the general population, higher NSCI scores are associated with spending more time looking at the night sky (Barnes and Passmore 2024). Second, we included a single‐item fear of the dark measure created for the current research, as people who fear the dark might avoid dark spaces.

Our main research question was how dark spaces (i.e., starry night sky and deep space) affect emotions, and in particular awe. We measured positive and negative affect to assess broader emotional experiences, which is consistent with previous research on the influence of natural environments on emotions (McMahan and Estes 2015). Both attention restoration theory (ART; Kaplan 1995) and the biophilia hypothesis (Kellert and Wilson 1995) suggest that people have an affinity for natural environments. ART suggests this is because such environments offer soft fascination (or easy engagement), which leads to natural environments being more pleasant and restorative. The biophilia hypothesis suggests positive affect emerges because we evolved a propensity for natural environments that helped us to survive. Consequently, both suggest positive emotional experiences for observing starry nights as the stars might provide soft fascination and have been used by cultures around the world for survival purposes (e.g., navigation), but because people did not evolve in deep space environments and they might be challenging to process given their novelty (making soft fascination less likely), we also anticipated a more negative experience from viewing deep space photographs than starry night images, but still more positive than urban environments which have no natural elements. This prediction might manifest after viewing deep space images as either increased negative affect or increased accommodation (i.e., the subscale of awe related to difficulty in making sense of what they are observing).

As a secondary goal, we assessed the perceived pleasantness and restoration of the photographs, as well as participants' willingness to hang up the photographs. These measures are related to ART (for systematic review, see Ohly et al. 2016) and are commonly used in ART research (e.g., White et al. 2014). They assess restoration and a broader liking of the environments rather than elicited affect.

## 2 Method

The current work involved a small face‐to‐face pilot study to test out the method because of the lack of research in the area, and a larger online study. In both cases, participants were exposed to photographs of deep space, stars, and urban environments using a within‐subjects design. Their levels of affect were assessed, perceived restoration, pleasantness of the photos, and willingness to hang them up, as well as their connection to the night sky and fear of the dark.

### 2.1 Participants

The pilot study consisted of 14 participants. We collected data for an academic term and then analyzed the results at the completion of the term rather than aiming for a particular sample size given that it was a pilot study primarily aimed at testing the method. The online version had 99 participants. Using G*Power (Faul et al. 2007), an a priori power analysis of a repeated measures ANOVA (i.e., the main analyses) with 0.95 power and a medium effect size of 0.25 suggested a sample of 43. Although the pilot study had larger effect sizes, we went with a medium effect size to be conservative. We also decided to exceed the sample size of 43 and collect data for an entire term because the online study might be less engaging than the face‐to‐face version, which might result in smaller effect sizes in the online version. The studies were identical except that the pilot study was conducted face‐to‐face using a large screen in a darkened room, while the online version was completed online via a survey. All other procedures were identical. Participants were recruited from a university campus in addition to public locations (e.g., in the city centre) to obtain a more diverse sample and avoid relying solely on a WEIRD sample of university students (Henrich et al. 2010).

### 2.2 Materials

#### 2.2.1 Stimuli

**FIGURE 1:**
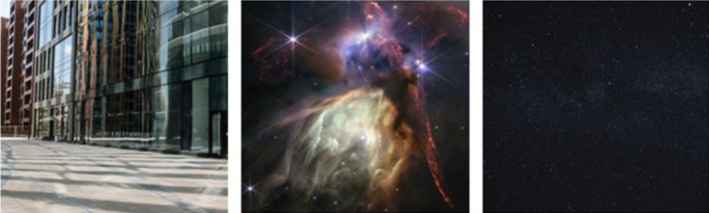
Examples of stimuli. From left to right: Photos of urban, deep space, and stars. Deep space photos are from the James Webb Space Telescope.

All stimuli were collected from online sources that were freely available (see Figure 1 for example stimuli). The deep space images were from the James Webb telescope. The selection criteria were that the photographs came from the James Webb telescope and were of different deep space phenomena (rather than different photographs of the same area). Star images were night sky images that contained stars that could be seen with the naked eye (i.e., what could be seen while looking up at night) without any other surroundings or clouds visible. Given the limited subject matter (i.e., stars at night), photographs were all similar in appearance, but as with the deep space images, we attempted to have photographs that were taken from different areas. The urban images were city spaces with no obvious vegetation or water present given that such features are known to affect emotions (McMahan and Estes 2015). Two research assistants confirmed that urban images met those requirements. There were 7 photos in each set, which were displayed on the screen for 20 s each with the following instructions: “Please take a moment to observe each photo and immerse yourself in the depicted environment.” After 20 s, the next photo automatically appeared.

#### 2.2.2 Dependent Variables

Awe was measured with the awe experience scale (AWE‐S; Yaden et al. 2019), which contains 6 subscales: time (e.g., “I noticed time slowing”), self‐loss (e.g., “I felt my sense of self shrink”), connectedness (e.g., “I felt closely connected to humanity”), vastness (e.g., “I felt in the presence of greatness”), physiological (‘I gasped’), and accommodation (e.g., “I found it was hard to comprehend the experience in full”). Ratings were on a 1 (strongly disagree) to 7 (strongly agree) scale. There are 30 items total with 5 per subscale. With over 180 citations at this time, it is a widely used measure.

Positive and negative affect were measured using the international positive and negative affect schedule short form (I‐PANAS‐SF; Thompson 2007). The 10‐item scale consists of 5 positive affect words (i.e., alert, inspired, determined, attentive, and active) and 5 negative affect words (i.e., upset, hostile, ashamed, nervous, and afraid) that are rated on a 1 (never) to 5 (always) scale on how participants felt.

Three questions that have been used in previous research on the influence of photos of natural environments were also included (e.g., White et al. 2014): “How would you rate the pleasantness of these images?”, “Overall, to what extent do you think that these images would be excellent for restoring your ability to concentrate or work effectively on a demanding project?”, and “How willing would you be to hang images of these environments in your room?” All items were rated on a 1 (not at all) to 10 (extremely) scale.

#### 2.2.3 Moderators and Demographics

The night sky connection index (NSCI) was used as a potential moderator (Barnes and Passmore 2024). It consists of 12 items rated on a 1 (strongly disagree) to 10 (strongly agree) scale. There are two subscales: connection (e.g., “I feel beauty in the night sky”) and protection (e.g., “I protect the night sky from light pollution”).

Also to be used as a moderator, a single‐item measure of fear of the dark that was created for the current research used a 1 (not at all) to 10 (extremely) scale: “How afraid are you of the dark?”

Demographic questions included their age, gender, ethnicity, whether they are currently a student, their highest level of education, and because recruitment included public locations where tourists might be present, which country they are from.

### 2.3 Procedure

After informed consent, participants viewed 7 photographs for 20 s each from one of the three within‐subjects conditions (randomly assigned). They then rated the set of images on the dependent variables. Once completed, the next set of photos was displayed, and the process continued until all three sets of photos were viewed and rated. Moderators and demographic questions followed, and the study ended with a debriefing. In the face‐to‐face version, the study was done individually in a darkened room while looking at a large screen TV that presented the stimuli. In the online version, the study was completed via an online survey. There was no compensation for participating. The study was anonymous and approved by a university research ethics board.

## 3 Results

### 3.1 Demographics

Full demographic information can be found in the Appendix A. Age, gender, and country are as follows. The pilot study: age (M = 25.50, SD = 6.16), gender (7% man, 79% woman, and 14% non‐binary/third gender), and country (100% Canada). And for the online study: age (M = 42.85, SD = 18.07), gender (32% man, 63% woman, 3% non‐binary/third gender, and 2% prefer not to say), and country (93% Canada, 4% USA, 1% Australia, 1% Georgia, and 1% New Zealand).

### 3.2 Main Analyses

Table 1 presents results of condition on AWE‐S (including subscales), positive affect, negative affect, pleasantness of images, willingness to hang up such images, and perceived restoration for both studies. Overall effects are reported in‐text; for differences between within‐subjects conditions, see Table 1.

**TABLE 1:** Means, standard deviations, and analysis of variance of urban, deep space, and stars on dependent variables.

Measure	Urban		Deep space		Stars		*F*	**η** ^2^
	M	SD	M	SD	M	SD		
AWE‐S full scale	2.77_a_ 2.63_a_	1.26 1.22	4.55_b_ 4.29_b_	1.22 1.37	4.02_b_ 3.97_b_	1.20 1.25	13.68*** 70.88***	0.51 0.43
Time	3.86_a_ 3.26_a_	1.59 1.57	4.87_a_ 4.50_b_	1.50 1.49	4.79_a_ 4.43_b_	1.20 1.50	2.90 27.33***	0.18 0.24
Self‐loss	2.84_a_ 2.49_a_	1.58 1.47	4.66_b_ 4.08_b_	1.77 1.92	3.99_ab_ 4.02_b_	1.65 1.62	6.38** 32.10***	0.33 0.30
Connectedness	2.40_a_ 2.29_a_	1.37 1.28	3.88_b_ 3.58_b_	1.59 1.88	3.45_ab_ 3.46_b_	1.50 1.69	4.97* 22.93***	0.29 0.23
Vastness	3.18_a_ 2.45_a_	1.44 1.44	5.54_b_ 5.18_b_	1.61 1.78	5.17_b_ 4.95_b_	1.37 1.66	15.86*** 92.04***	0.55 0.53
Physiological	1.38_a_ 1.58_a_	0.68 1.03	2.77_a_ 3.43_b_	1.56 1.65	2.47_a_ 2.55_c_	1.44 1.60	6.39** 38.54***	0.37 0.37
Accommodation	2.16_a_ 2.51_a_	1.46 1.51	5.42_b_ 4.53_b_	1.56 1.75	4.27_b_ 3.68_c_	2.04 1.64	15.40*** 40.37***	0.58 0.35
Pleasant	4.07_a_ 4.67_a_	1.90 2.03	7.29_b_ 7.10_b_	2.09 2.32	7.14_b_ 7.05_b_	2.60 1.90	11.51*** 47.54***	0.47 0.34
Restoration	3.64_a_ 3.56_a_	1.86 2.14	5.79_b_ 4.88_b_	2.01 2.48	6.36_b_ 5.61_c_	2.02 2.46	8.41** 22.81***	0.39 0.22
Willingness	3.75_a_ 3.77_a_	2.63 2.52	6.75_a_ 5.94_b_	2.70 3.03	6.58_a_ 5.84_b_	3.00 2.78	4.40* 19.66***	0.29 0.20
Positive affect	1.87_a_ 2.01_a_	0.76 0.84	2.55_a_ 2.71_b_	0.97 1.03	2.24_a_ 2.71_b_	0.97 1.10	2.99 27.03***	0.20 0.25
Negative affect	1.16_a_ 1.39_a_	0.23 0.56	1.44_a_ 1.50_a_	0.40 0.85	1.28_a_ 1.28_a_	0.70 0.48	1.26 2.90	0.11 0.05

*Note:* Within each measure, the first row represents face‐to‐face study while the second row represents online study. Means not sharing subscripts within a row differ significantly with Bonferroni adjustment. *p < 0.05; **p < 0.01; ***p < 0.001 for F‐test.

#### 3.2.1 Awe

Reliabilities for awe and its subscales were good for both studies (all αs > 0.80; see Appendix A for full results).

For the face‐to‐face pilot study, a repeated measures analysis of variance (ANOVA) of condition (urban vs. space vs. stars) was run on the overall awe scale, which was significant, *F*(2, 26) = 13.68, *p* < 0.001, partial η^2^ = 0.51. For the subscales, time was ns, *F*(2, 26) = 2.90, *p* = 0.07, partial η^2^ = 0.18; self‐loss was significant, *F*(2, 26) = 6.38, *p* = 0.006, partial η^2^ = 0.33; connectedness was significant, *F*(2, 24) = 4.97, *p* = 0.02, partial η^2^ = 0.29; vastness was significant, *F*(2, 26) = 15.86, *p* < 0.001, partial η^2^ = 0.55; physiological was significant, *F*(2, 22) = 6.39, *p* = 0.006, partial η^2^ = 0.37; and accommodation was significant, *F*(2, 22) = 15.40, *p* < 0.001, partial η^2^ = 0.58.

For the online study, the overall awe scale was significant, *F*(2, 188) = 70.88, *p* < 0.001, partial η^2^ = 0.43. All subscales were statistically significant: time, *F*(2, 176) = 27.33, *p* < 0.001, partial η^2^ = 0.24; self‐loss, *F*(2, 150) = 32.10, *p* < 0.001, partial η^2^ = 0.30; connectedness, *F*(2, 150) = 22.93, *p* < 0.001, partial η^2^ = 0.23; vastness, *F*(2, 162) = 92.04, *p* < 0.001, partial η^2^ = 0.53; physiological, *F*(2, 132) = 38.54, *p* < 0.001, partial η^2^ = 0.37; and accommodation, *F*(2, 148) = 40.37, *p* < 0.001, partial η^2^ = 0.35.

These results suggest that stars, and in particular deep space, induce greater feelings of awe compared to urban environments, with larger effects for vastness and accommodation subscales.

#### 3.2.2 Positive and Negative Affect

Reliabilities for positive affect were good. For the face‐to‐face study within urban, space, and stars conditions, respectively, αs = 0.82, 0.90, and 0.89. For the online study within urban, space, and stars conditions, respectively, αs = 0.81, 0.86, and 0.88.

Reliabilities for negative affect were low in the face‐to‐face study, but acceptable in the online study. For the face‐to‐face study within urban, space, and stars conditions, respectively, αs = 0.20, 0.55, and 0.63. For the online study within urban, space, and stars conditions, respectively, αs = 78, 0.88, and 0.63. Consequently, negative affect results in the face‐to‐face study should be interpreted with caution.

For the face‐to‐face study, there were no significant effects on positive affect, *F*(2, 24) = 2.99, *p* = 0.07, partial η^2^ = 0.20, or negative affect, *F*(2, 20) = 1.26, *p* = 0.31, partial η^2^ = 0.11. However, for the online version, there was a significant effect on positive affect, *F*(2, 162) = 27.03, *p* < 0.001, partial η^2^ = 0.25, but negative affect just failed to reach statistical significance, *F*(2, 112) = 2.90, *p* = 0.06, partial η^2^ = 0.05.

Consequently, mixed results were found on affect, with stars and deep space sometimes inducing greater positive affect, but no effects on negative affect were found.

#### 3.2.3 Pleasant, Restoration, and Willingness

The single‐item measures of how pleasant the images were, how restorative the images were, and their willingness to put the images up in their room were assessed.

For the face‐to‐face study, there were significant effects on how pleasant the images were, *F*(2, 26) = 11.51, *p* < 0.001, partial η^2^ = 0.47, their perceived restoration, *F*(2, 26) = 8.41, *p* = 0.002, partial η^2^ = 0.39, and willingness to hang the images in their room, *F*(2, 22) = 4.40, *p* = 0.03, partial η^2^ = 0.29.

Similarly for the online study, there were significant effects on how pleasant the images were, *F*(2, 184) = 47.54, *p* < 0.001, partial η^2^ = 0.34, their perceived restoration, *F*(2, 162) = 22.81, *p* < 0.001, partial η^2^ = 0.22, and willingness to hang the images in their room, *F*(2, 156) = 19.66, *p* < 0.001, partial η^2^ = 0.20.

Results suggest that deep space and stars photos were more pleasant than urban ones, and participants were more willing to hang up such photos. However, although both deep space and stars were perceived as more restorative than urban photos, stars were perceived as the most restorative. This last finding is in contrast to much of what has been reviewed thus far, where deep space is often the one scoring higher of the two. That is, although deep space might induce greater feelings of awe than stars, it is the stars that are perceived as more restorative.

### 3.3 Moderators

Reliabilities for NSCI and its subscales in the online study were good, for full scale, connection, and protection, respectively, αs = 0.94, 0.94, and 0.73.

Only interactions and main effects of the moderators are reported here; for the full results (including Ms and SDs), please see the Appendix A. Potential moderators were tested in the larger online study by creating median splits and inserting them into the ANOVAs as between‐subjects factors, with conditions still as within‐subjects factors. Using NSCI and its two subscales (connection and protection) on the full awe scale, there were no interactions and only main effects of the full NSCI and connection subscale: full scale, *F*(1, 93) = 4.42, *p* = 0.04, partial η^2^ = 0.05, and connection subscale, *F*(1, 93) = 15.59, *p* < 0.001, partial η^2^ = 0.14. Results indicated that the higher their overall NSCI and connection subscale scores, the higher their awe ratings.

Similarly, for positive affect there was no interaction, but there was a main effect of NSCI, *F*(1, 80) = 5.91, *p* = 0.02, partial η^2^ = 0.07. There was also a main effect using the connection subscale, *F*(1, 80) = 7.10, *p* = 0.01, partial η^2^ = 0.08. Results indicate that those with higher overall NCSI and connection subscale scores experience more positive affect.

For negative affect, there were no significant interactions or main effects of the NSCI or its subscales.

For pleasantness of the images, there was a main effect of NSCI, *F*(1, 91) = 6.48, *p* = 0.01, partial η^2^ = 0.07, as well as the connection subscale, *F*(1, 91) = 5.40, *p* = 0.02, partial η^2^ = 0.06. Results indicate that those higher in NSCI and its connection subscale experience the photos as more pleasant.

For perceived restoration, there was a significant interaction, *F*(2, 160) = 3.27, *p* = 0.04, partial η^2^ = 0.04, as well as a main effect of NSCI, *F*(1, 80) = 4.92, *p* = 0.03, partial η^2^ = 0.06 (see Appendix A for Figure). Results suggest that while NSCI had no effect on urban photos, it did on space and stars photos, with those scoring higher on NSCI rating them as more restorative.

Similarly, for willingness to hang an image in their room, there was a significant interaction with NSCI, *F*(2, 154) = 4.25, *p* = 0.02, partial η^2^ = 0.05, and also interactions with the protection subscale, *F*(2, 150) = 3.43, *p* = 0.04, partial η^2^ = 0.05, and connection subscale, *F*(2, 154) = 7.09, *p* = 0.001, partial η^2^ = 0.08 (see Appendix A for Figures). Results suggest that those higher in NSCI and its subscales report a greater willingness to hang an image of space and stars in their room compared to those who score lower.

Next, assessing fear of the dark as a potential moderator utilized the same strategy by conducting a median split and added it to ANOVAs on the various DVs. There was a main effect on positive affect, *F*(1, 77) = 4.85, *p* = 0.03, partial η^2^ = 0.06, which indicated that, perhaps surprisingly, higher fear of the dark was associated with more positive affect. There was also a main effect on negative affect, *F*(1, 52) = 6.99, *p* = 0.01, partial η^2^ = 0.12, which indicated that higher fear of the dark was associated with more negative affect. No other significant effects emerged.

## 4 Discussion

The current work adds to our understanding of how dark spaces influence emotions. First, it used highly detailed photos from the James Webb telescope. Understanding their influence is worthwhile given that people are exposed to such photos through the media. Indeed, their influence is not identical to other dark spaces like viewing stars (e.g., stars are more restorative than space, but space induces more positive affect than stars).

Second, it uses a measure of awe with several subscales (Yaden et al. 2019), which gives a more nuanced understanding of how awe is influenced. The strongest effects were observed with the vastness and accommodation subscales, while time and connectedness showed weaker effects. Although the influence of deep space and stars was generally similar, they also weren't identical, with deep space typically scoring higher of the two. These findings suggest that different dark spaces do not have uniform effects on awe experiences. In terms of the accommodation subscale, it might be perceived as being a relatively negative experience given that items involve difficulty comprehending what they are observing (e.g., “I found it hard to comprehend the experience in full” and “I felt challenged to understand the experience”). Accommodation was higher for deep space images than for starry night ones in the online study, which is consistent with our prediction that deep space will be a more negative experience—though there were no differences in negative affect. In addition, we replicated previous work (Bell et al. 2014) demonstrating that starry night images lead to a loss of time and diminished sense of self (as measured by the time and self‐diminished subscales, respectively), but we extend these findings by demonstrating that similar effects occur for deep space images as well. In general, the results suggest that it is important to measure awe with a multifaceted approach.

Third, although limited effects were found for positive and negative affect, with positive affect in the larger online study being the only significant effect (in the predicted direction), this is somewhat consistent with research on other natural environments (e.g., green spaces), which often find inconsistent effects on negative affect, but more consistent effects on positive affect (Song et al. 2022). However, before any conclusions are drawn, findings should first be replicated.

Fourth, moderation was tested with a recently developed connection to the night sky measure (Barnes and Passmore 2024). However, such a connection seems only broadly relevant to emotional experiences like awe and positive affect (with higher connecting related to higher positive affect and awe across stimuli), but for perceived restoration and willingness to be around such imagery, such a connection seems relevant to deep space and stars imagery (with higher connecting leading to higher restoration and willingness to put up the images, but only for deep space and star images). Conversely, fear of the dark was associated with positive and negative affect in general, but not the other measures. It is not clear why such differences emerged, so this is an area for future research.

Several limitations are worth mentioning. First, the number of emotions tested was limited. We focused on awe given the link with previous research (Bell et al. 2014), but also measured positive and negative affect. However, measuring positive and negative affect broadly—though consistent with previous research (McMahan and Estes 2015)—might hide effects on specific emotions. Assessing a larger number of specific affective states is an avenue for future research.

Another limitation is that image size and quality in the online study was difficult to control given the likely variability in devices used to complete the study (e.g., Smartphones, laptops). However, we pre‐tested the online survey on both Smartphones and laptop/desktop computers, and size and quality did not seem to be an issue. Importantly, effects still emerged, and if image size and quality were an issue, effect sizes would likely be diminished as a result, which might suggest effect sizes are underestimated here. Nonetheless, future research might more closely control for such variation in stimuli presentation.

The demographics of the two samples varied somewhat from each other, which might have influenced results. One difference is that 100% of participants in the face‐to‐face study were Canadian, while 93% were in the online study. The majority were Canadian across samples, but it remains a possibility that sample differences affected results. Although our predictions are not dependent on cultural background (i.e., they are assumed to be more universal; Kellert and Wilson 1995), future research should more explicitly test for cross‐cultural differences and assess the influence of other demographic variables.

A final limitation relates to the control condition. Given that the majority of the world's population lives in urban areas, which is particularly true in Canada where our samples were drawn (Our World in Data 2025), we employed urban photographs as a baseline control condition. Although this gives us a comparison with a baseline, it also means that it is unclear how dark spaces compare to other natural environments, such as green spaces, which are known to have positive effects (McMahan and Estes 2015). We did not include a green space condition to limit the duration of the study to avoid fatiguing or boring participants, but this is an important area for future research.

In summary, photographs of deep space and stars can have positive effects on people, but such effects are also nuanced and not uniform.

## Author Contributions

J.P.M., M.P., and K.T. were involved in study design and drafting the article; M.P. and K.T. collected the data, and J.P.M. analysed the data.

## Funding

The authors have nothing to report.

## Ethics Statement

All procedures performed in studies involving human participants were in accordance with the ethical standards of the institutional research committee at [anonymized for review] and with the 1964 Helsinki Declaration and its later amendments or comparable ethical standards.

## Consent

Informed consent was obtained from all individual adult participants included in the study.

## Conflicts of Interest

The authors declare no conflicts of interest.

## Data Availability

The data that support the findings of this study are openly available in OSF at https://osf.io/hkgm4/?view_only=c03d667b7bea43cd89fb618058044003.

## Appendix A

### Demographics

Demographics for the pilot study were as follows: age (M = 25.50, SD = 6.16), gender (7% man, 79% woman, and 14% non‐binary/third gender), country (100% Canada), ethnicity (64% European, 14% Indigenous, 7% Asian, 7% prefer not to say, and 7% mixed), university student (86% yes, 14% no), and education (64% some post secondary but no degree, 21% post secondary with degree, 7% graduate or professional degree, and 7% secondary/high‐school). And for the online study: age (M = 42.85, SD = 18.07), gender (32% man, 63% woman, 3% non‐binary/third gender, and 2% prefer not to say), country (93% Canada, 4% USA, 1% Australia, 1% Georgia, and 1% New Zealand), ethnicity (66% European, 9% other, 7% Asian, 6% prefer not to say, 5% mixed, 3% Middle Eastern, 2% Indigenous, 1% Latin American), university student (23% yes, 77% no), and education (41% some post secondary but no degree, 32% post secondary with degree, 16% graduate or professional degree, 8% secondary/high‐school, and 2% prefer not to say).

### Reliabilities

For the face‐to‐face study within urban, space, and stars conditions, respectively, for the full scale, αs = 0.94, 0.97, & 0.96; for time, αs = 0.95, 0.93, & 0.93; for self‐loss, αs = 0.96, 0.95, & 0.97; for connectedness, αs = 0.92, 0.93, & 0.92; for vastness, αs = 0.87, 0.89, & 0.81; for physiological, αs = 0.80, 0.92, & 0.83; and for accommodation, αs = 0.89, 0.94, & 0.96. For the online study within urban, space, and stars conditions, respectively, for the full scale, αs = 0.97, 0.98, & 0.96; for time, αs = 0.95, 0.94, & 0.94; for self‐loss, αs = 0.95, 0.97, & 0.94; for connectedness, αs = 0.92, 0.97, & 0.97; for vastness, αs = 0.91, 0.96, & 0.94; for physiological, αs = 0.96, 0.88, & 0.96; and for accommodation, αs = 0.93, 0.94, & 0.92.

### Moderators

With the full NSCI scale on the full AWE scale, there was no interaction, *F*(2, 186) = 0.55, *p* = 0.58, partial η^2^ = 0.01. There were main effects of condition, *F*(2, 186) = 70.40, *p* < 0.001, partial η^2^ = 0.34, and NSCI, *F*(1, 93) = 4.42, *p* = 0.04, partial η^2^ = 0.05. Means with standard deviations in brackets for low versus high NSCI, respectively, for each condition are as follows. Urban: 2.51 (1.28) & 2.75 (1.16). Deep space: 4.03 (1.56) & 4.55 (1.13). Stars: 3.72 (1.19) & 4.21 (1.27).

With the NSCI connection subscale, there was no interaction, *F*(2, 186) = 0.02, *p* = 0.98, partial η^2^ = 0.00. There were main effects of condition, *F*(2, 186) = 70.10, *p* < 0.001, partial η^2^ = 0.43, and NSCI connection, *F*(1, 93) = 15.59, *p* < 0.001, partial η^2^ = 0.14. Means with standard deviations in brackets for low versus high NSCI connection, respectively, for each condition are as follows. Urban: 2.24 (1.16) & 3.00 (1.17). Deep space: 3.91 (1.54) & 4.66 (1.09). Stars: 3.61 (1.10) & 4.31 (1.30).

With the NSCI protection subscale, there was no interaction, *F*(2, 176) = 1.05, *p* = 0.35, partial η^2^ = 0.01. There was a main effect of condition, *F*(2, 176) = 70.06, *p* < 0.001, partial η^2^ = 0.44, but not for NSCI protection, *F*(1, 88) = 0.61, *p* = 0.44, partial η^2^ = 0.01. Means with standard deviations in brackets for low versus high NSCI protection, respectively, for each condition are as follows. Urban: 2.68 (1.26) and 2.62 (1.18). Deep space: 4.22 (1.43) and 4.38 (1.28). Stars: 3.82 (1.23) and 4.21 (1.26).

With the fear of the dark measure, there was no interaction, *F*(2, 174) = 0.70, *p* = 0.50, partial η^2^ = 0.01. There was a main effect of condition, *F*(2, 174) = 68.96, *p* < 0.001, partial η^2^ = 0.44, but not fear of dark, *F*(1, 87) = 2.76, *p* = 0.10, partial η^2^ = 0.03. Means with standard deviations in brackets for low versus high fear of dark, respectively, for each condition are as follows. Urban: 2.53 (1.16) and 2.77 (1.33). Deep space: 4.06 (1.51) and 4.61 (1.01). Stars: 3.92 (1.31) and 4.15 (1.12).

With the full NSCI scale on positive affect, there was no interaction, *F*(2, 160) = 2.55, *p* = 0.08, partial η^2^ = 0.03. There were main effects of condition, *F*(2, 160) = 27.15, *p* < 0.001, partial η^2^ = 0.25, and NSCI, *F*(1, 80) = 5.91, *p* = 0.02, partial η^2^ = 0.07. Means with standard deviations in brackets for low versus high NSCI, respectively, for each condition are as follows. Urban: 1.93 (0.74) & 2.09 (0.91). Deep space: 2.47 (0.96) & 2.94 (1.05). Stars: 2.38 (0.95) & 3.03 (1.15).

With the NSCI connection subscale, there was no interaction, *F*(2, 160) = 1.26, *p* = 0.29, partial η^2^ = 0.02. There were main effects of condition, *F*(2, 160) = 27.38, *p* < 0.001, partial η^2^ = 0.26, and NSCI connection, *F*(1, 80) = 7.10, *p* < 0.01, partial η^2^ = 0.08. Means with standard deviations in brackets for low versus high NSCI connection, respectively, for each condition are as follows. Urban: 1.89 (0.72) and 2.15 (0.93). Deep space: 2.45 (0.96) and 2.98 (1.04). Stars: 2.43 (0.96) and 3.01 (1.16).

With the NSCI protection subscale, there was no interaction, *F*(2, 156) = 0.21, *p* = 0.82, partial η^2^ = 0.003. There was a main effect of condition, *F*(2, 156) = 26.49, *p* < 0.001, partial η^2^ = 0.25, but not for NSCI protection, *F*(1, 78) = 1.01, *p* = 0.32, partial η^2^ = 0.01. Means with standard deviations in brackets for low versus high NSCI protection, respectively, for each condition are as follows. Urban: 1.92 (0.79) & 2.09 (0.90). Deep space: 2.66 (1.03) & 2.78 (1.05). Stars: 2.57 (1.04) & 2.83 (1.18).

With the fear of the dark measure, there was no interaction, *F*(2, 154) = 0.87, *p* = 0.42, partial η^2^ = 0.01. There were main effects of condition, *F*(2, 154) = 26.60, *p* < 0.001, partial η^2^ = 0.26, and fear of dark, *F*(1, 77) = 4.85, *p* = 0.03, partial η^2^ = 0.06. Means with standard deviations in brackets for low versus high fear of dark, respectively, for each condition are as follows. Urban: 1.92 (0.84) & 2.15 (0.84). Deep space: 2.48 (1.07) & 2.98 (0.94). Stars: 2.53 (1.11) & 3.00 (1.10).

With the full NSCI scale on negative affect, there was no interaction, *F*(2, 110) = 0.95, *p* = 0.39, partial η^2^ = 0.02. There were no main effects of condition, *F*(2, 110) = 2.89, *p* = 0.06, partial η^2^ = 0.05, and NSCI, *F*(1, 55) = 0.01, *p* = 0.92, partial η^2^ = 0.00. Means with standard deviations in brackets for low versus high NSCI, respectively, for each condition are as follows. Urban: 1.31 (0.61) & 1.47 (0.51). Deep space: 1.53 (0.90) & 1.47 (0.83). Stars: 1.31 (0.46) & 1.25 (0.50).

With the NSCI connection subscale, there was no interaction, *F*(2, 110) = 0.92, *p* = 0.40, partial η^2^ = 0.02. There were no main effects of condition, *F*(2, 110) = 2.91, *p* = 0.06, partial η^2^ = 0.05, and NSCI connection, *F*(1, 55) = 0.14, *p* = 0.71, partial η^2^ = 0.003. Means with standard deviations in brackets for low versus high NSCI connection, respectively, for each condition are as follows. Urban: 1.30 (0.54) and 1.49 (0.57). Deep space: 1.49 (0.87) and 1.50 (0.85). Stars: 1.30 (0.52) and 1.25 (0.44).

With the NSCI protection subscale, there was no interaction, *F*(2, 108) = 0.58, *p* = 0.56, partial η^2^ = 0.01. There were no main effects of condition, *F*(2, 108) = 2.13, *p* = 0.12, partial η^2^ = 0.04, and NSCI protection, *F*(1, 54) = 0.16, *p* = 0.69, partial η^2^ = 0.003. Means with standard deviations in brackets for low versus high NSCI protection, respectively, for each condition are as follows. Urban: 1.30 (0.53) & 1.43 (0.51). Deep space: 1.48 (0.94) & 1.43 (0.66). Stars: 1.23 (0.42) & 1.31 (0.54).

With the fear of the dark measure, there was no interaction, *F*(2, 104) = 0.33, *p* = 0.69, partial η^2^ = 0.01. There was no main effect of condition, *F*(2, 104) = 2.07, *p* = 0.13, partial η^2^ = 0.04, and a significant effect of fear of dark, *F*(1, 52) = 6.99, *p* = 0.01, partial η^2^ = 0.12. Means with standard deviations in brackets for low versus high fear of dark, respectively, for each condition are as follows. Urban: 1.21 (0.44) & 1.57 (0.59). Deep space: 1.27 (0.57) & 1.66 (0.95). Stars: 1.16 (0.28) & 1.40 (0.63).

With the full NSCI scale on pleasantness, there was no interaction, *F*(2, 182) = 0.40, *p* = 0.67, partial η^2^ = 0.004. There were main effects of condition, *F*(2, 182) = 47.15, *p* < 0.001, partial η^2^ = 0.34, and NSCI, *F*(1, 91) = 6.48, *p* = 0.01, partial η^2^ = 0.07. Means with standard deviations in brackets for low versus high NSCI, respectively, for each condition are as follows. Urban: 4.44 (2.13) & 4.89 (1.93). Deep space: 6.61 (2.34) & 7.57 (2.22). Stars: 6.72 (1.59) & 7.38 (2.12).

With the NSCI connection subscale, there was no interaction, *F*(2, 182) = 0.64, *p* = 0.53, partial η^2^ = 0.01. There were main effects of condition, *F*(2, 182) = 47.34, *p* < 0.001, partial η^2^ = 0.34, and NSCI connection, *F*(1, 91) = 5.40, *p* = 0.02, partial η^2^ = 0.06. Means with standard deviations in brackets for low versus high NSCI connection, respectively, for each condition are as follows. Urban: 4.35 (2.08) and 4.98 (1.95). Deep space: 6.61 (2.43) and 7.57 (2.13). Stars: 6.89 (1.61) and 7.21 (2.15).

With the NSCI protection subscale, there was no interaction, *F*(2, 174) = 1.66, *p* = 0.19, partial η^2^ = 0.02. There was a main effect of condition, *F*(2, 174) = 49.11, *p* < 0.001, partial η^2^ = 0.36, but not NSCI protection, *F*(1, 87) = 1.23, *p* = 0.27, partial η^2^ = 0.01. Means with standard deviations in brackets for low versus high NSCI protection, respectively, for each condition are as follows. Urban: 4.56 (2.09) & 4.77 (2.00). Deep space: 6.78 (2.26) & 7.66 (2.15). Stars: 7.13 (1.73) & 6.98 (2.13).

With the fear of the dark measure, there was no interaction, *F*(2, 170) = 0.08, *p* = 0.92, partial η^2^ = 0.001. There was a main effect of condition, *F*(2, 170) = 44.02, *p* < 0.001, partial η^2^ = 0.34, but not fear of dark, *F*(1, 85) = 0.001, *p* = 0.97, partial η^2^ = 0.000. Means with standard deviations in brackets for low versus high fear of dark, respectively, for each condition are as follows. Urban: 4.69 (2.15) and 4.74 (1.86). Deep space: 7.20 (2.11) and 7.32 (2.45). Stars: 7.12 (1.86) and 7.00 (2.07).

With the full NSCI scale on perceived restoration, there was a significant interaction, *F*(2, 160) = 3.27, *p* = 0.04, partial η^2^ = 0.04. There were main effects of condition, *F*(2, 160) = 22.76, *p* < 0.001, partial η^2^ = 0.22, and NSCI, *F*(1, 80) = 4.92, *p* = 0.03, partial η^2^ = 0.06. Means with standard deviations in brackets for low versus high NSCI, respectively, for each condition are as follows. Urban: 3.56 (2.15) & 3.56 (2.15). Deep space: 4.08 (2.23) & 5.60 (2.49). Stars: 5.10 (2.40) & 6.07 (2.44).

With the NSCI connection subscale, there was no interaction, *F*(2, 160) = 2.18, *p* = 0.18, partial η^2^ = 0.03. There was a main effect of condition, *F*(2, 160) = 23.14, *p* < 0.001, partial η^2^ = 0.22, but not NSCI connection, *F*(1, 80) = 3.97, *p* = 0.05, partial η^2^ = 0.05. Means with standard deviations in brackets for low versus high NSCI connection, respectively, for each condition are as follows. Urban: 3.48 (2.19) & 3.63 (2.11). Deep space: 4.17 (2.25) & 5.59 (2.52). Stars: 5.27 (2.52) & 5.95 (2.38).

With the NSCI protection subscale, there was no interaction, *F*(2, 158) = 1.62, *p* = 0.20, partial η^2^ = 0.02. There was a main effect of condition, *F*(2, 158) = 23.08, *p* < 0.001, partial η^2^ = 0.23, but not NSCI protection, *F*(1, 79) = 0.01, *p* = 0.92, partial η^2^ = 0.000. Means with standard deviations in brackets for low versus high NSCI protection, respectively, for each condition are as follows. Urban: 3.73 (2.23) and 3.35 (2.07). Deep space: 4.59 (2.48) and 5.25 (2.45). Stars: 5.66 (2.28) and 5.50 (2.66).

With the fear of the dark measure, there was no interaction, *F*(2, 150) = 0.76, *p* = 0.47, partial η^2^ = 0.01. There was a main effect of condition, *F*(2, 150) = 20.14, *p* < 0.001, partial η^2^ = 0.21, but not fear of dark, *F*(1, 75) = 0.66, *p* = 0.42, partial η^2^ = 0.01. Means with standard deviations in brackets for low versus high fear of dark, respectively, for each condition are as follows. Urban: 3.23 (2.03) & 4.03 (2.34). Deep space: 4.96 (2.55) & 5.00 (2.42). Stars: 5.60 (2.65) & 5.77 (2.34).

With the full NSCI scale on willingness to hang up photo, there was a significant interaction, *F*(2, 154) = 4.25, *p* = 0.02, partial η^2^ = 0.05. There was a main effect of condition, *F*(2, 154) = 20.24, *p* < 0.001, partial η^2^ = 0.21, but not NSCI, *F*(1, 77) = 1.11, *p* = 0.30, partial η^2^ = 0.01. Means with standard deviations in brackets for low versus high NSCI, respectively, for each condition are as follows. Urban: 4.18 (2.75) & 3.38 (2.23). Deep space: 5.28 (3.00) & 6.58 (2.95). Stars: 5.38 (2.68) & 6.28 (2.85).

With the NSCI connection subscale, there was a significant interaction, *F*(2, 154) = 7.09, *p* = 0.001, partial η^2^ = 0.08. There was a main effect of condition, *F*(2, 154) = 20.90, *p* < 0.001, partial η^2^ = 0.21, but not NSCI connection, *F*(1, 77) = 0.10, *p* = 0.75, partial η^2^ = 0.001. Means with standard deviations in brackets for low versus high NSCI connection, respectively, for each condition are as follows. Urban: 4.46 (2.61) & 3.10 (2.25). Deep space: 5.21 (3.00) & 6.65 (2.91). Stars: 5.67 (2.74) & 6.00 (2.86).

With the NSCI protection subscale, there was a significant interaction, *F*(2, 150) = 3.43, *p* = 0.04, partial η^2^ = 0.04. There was a main effect of condition, *F*(2, 150) = 18.91, *p* < 0.001, partial η^2^ = 0.20, but not NSCI protection, *F*(1, 75) = 0.31, *p* = 0.58, partial η^2^ = 0.004. Means with standard deviations in brackets for low versus high NSCI protection, respectively, for each condition are as follows: Urban: 4.26 (2.66) & 3.34 (2.33). Deep space: 5.44 (3.04) & 6.45 (3.05). Stars: 5.49 (2.57) & 6.13 (2.96).

With the fear of the dark measure, there was no interaction, *F*(2, 148) = 0.17, *p* = 0.85, partial η^2^ = 0.002. There was a main effect of condition, *F*(2, 148) = 17.88, *p* < 0.001, partial η^2^ = 0.20, but not fear of dark, *F*(1, 74) = 1.18, *p* = 0.28, partial η^2^ = 0.02. Means with standard deviations in brackets for low versus high fear of dark, respectively, for each condition are as follows. Urban: 3.95 (2.32) and 3.59 (2.84). Deep space: 6.09 (2.96) and 5.75 (3.12). Stars: 6.23 (2.84) and 5.47 (2.72).

Figures of significant interactions.

Interaction between NSCI (full) and condition on perceived restoration.

Interaction between NSCI (full) and condition on willingness to hang image in room.

Interaction between NSCI (protection subscale) and condition on willingness to hang image in room.

Interaction between NSCI (connection subscale) and condition on willingness to hang image in room.
